# Mononeuropathy due to Entrapment of Dorsal Cutaneous Peroneal Nerve: Clinical, Electrophysiological, and Imaging Findings

**DOI:** 10.7759/cureus.3350

**Published:** 2018-09-24

**Authors:** Nidhi Shankar Kikkeri, Ragha Chaitanya Sakuru, Ruben Ngnitewe Massa'a, Pradeep C Bollu, Raghav Govindarajan

**Affiliations:** 1 Neurology, University of Missouri, Columbia, USA

**Keywords:** electromyography, nerve conduction study, ultrasound, paresthesias

## Abstract

Peroneal neuropathy is the most common mononeuropathy encountered in the lower extremities. Isolated injuries to the dorsal cutaneous peroneal nerve (DCPN) are uncommon, and most of the reported cases are due to trauma or iatrogenic causes. We report a case of a middle-aged woman who presented with a nine-month history of tingling sensation over the dorsum of her left foot with normal electromyography (EMG) findings and was subsequently diagnosed with entrapment of the DCPN at the ankle by ultrasonographic examination.

## Introduction

Peroneal neuropathy is the most common entrapment neuropathy in the lower extremity and accounts for 15% of all mononeuropathies in adults [1]. Peroneal nerve injury can result in numbness, tingling or weakness manifesting as a foot drop. The type of symptoms and the areas affected depend on the site of nerve injury. The common etiologies include fractures, traction injuries, compression, prolonged squatting or iatrogenic causes [1]. Here we report the case of a middle-aged woman who presented with paresthesias over the dorsum of the left foot with normal electromyography (EMG) findings and was subsequently diagnosed with entrapment of the dorsal cutaneous peroneal nerve (DCPN) at the ankle by neuromuscular ultrasound.

## Case presentation

A 58-year-old female patient with a past medical history significant for a migraine headache was referred to the neurology clinic for complaints of paresthesias in the left foot for nine months. Paresthesias were described as tingling that was precipitated every time she tried to put her right foot on top of the left foot. She further described that she would feel ‘zapp’-like sensations over last two toes on the dorsum of the left foot. She denied associated weakness or similar complaints in the right foot. She also denied a prior history of diabetes mellitus, ankle trauma, sprains, arthroscopies or ankle surgeries.

Neurological examination was remarkable for precipitation of paresthesias on the dorsum of the left foot with percussion on the dorsum of the left ankle joint (positive tinel sign). Sensations were intact to gross touch and pinprick. There was no evidence of a foot drop, and the motor strength was 5/5 in all extremities. Deep tendon reflexes were intact. 

**Figure 1:**
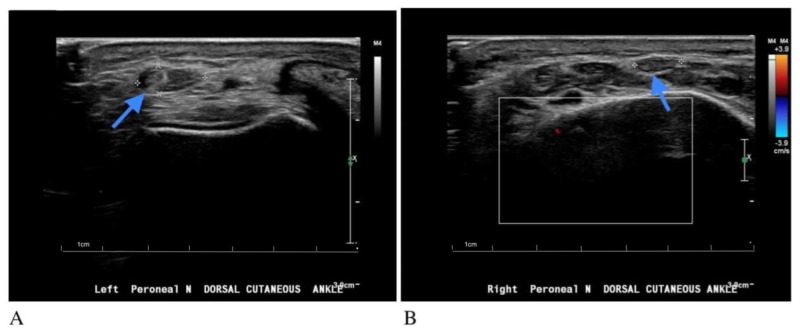
Images of neuromuscular limited ultrasound (Phillips, Affiniti 50G) using high frequency transducer (7-14 MHz). Image A shows enlarged left DCPN with cross-sectional area of 27.88 mm^2^ as compared to Image B which shows right DCPN with cross-sectional area of 10.8 mm^2^. DCPN: Dorsal cutaneous peroneal nerve.

Initial laboratory workup revealed white blood cell (WBC) count of 4.89 x 10^3^/nl (normal: 3.5-10.5 x 10^3^/nl), hemoglobin of 13.4 g/dL (normal: 12-15.5 g/dL) and mean corpuscular volume of 93.5 fL (normal: 81.6-98.3 fL). Thyroid stimulation hormone (TSH) level was 1.720 micIU/mL (normal: 0.270-4.200 micIU/mL). Blood urea nitrogen and serum creatinine levels were 17 mg/dL (normal: 6-20 mg/dL) and 0.79 mg/dL (normal: 0.5-1.2 mg/dL), respectively. Vitamin B12 level was 597 pg/mL (normal: 230-1245 pg/mL). Electrodiagnostic studies were done and reported to be normal with no evidence of neuropathy, radiculopathy or tarsal tunnel syndrome. Nerve conduction study (NCS) of the medial and lateral branches of the right and left superficial peroneal nerves (SPN) showed no significant side-to-side difference in antidromic sensory responses, as shown in Table 1. The patient then underwent a neuromuscular ultrasound evaluation of bilateral DCPNs at the level of the ankle. The study revealed a significantly enlarged left DCPN with a cross-sectional area of 27.88 mm^2^ at the level of the ankle, as compared to the cross-sectional area of the right DCPN, which was 10.8 mm^2^, as seen in Figure 1.

**Table 1:** Sensory nerve conduction study of branches of superficial peroneal nerve showing no evidence of significant side-to-side differences in peak latency and amplitude.

Superficial Peroneal Nerve	Branch	Peak Latency (ms)	Amplitude (microV)
Left	Medial	3.5	4.6
	Lateral	3.8	5.5
Right	Medial	3.6	5.3
	Lateral	3.4	6.8

## Discussion

Peroneal neuropathy is the third most common focal neuropathy after median and ulnar neuropathies [1-2]. Men appear to be more frequently affected than women [3]. Here we have discussed the anatomical course of the common peroneal nerve and its branches for a better understanding of the localization of lesions. The sciatic nerve divides just above the popliteal fossa into the common peroneal nerve (CPN) and the tibial nerve [4]. The superficial peroneal nerve (SPN) arises from the CPN [5] in the proximal leg and travels distally through its lateral compartment. After innervating the peroneus longus and peroneus brevis muscles, the SPN provides sensations to the lower two-thirds of the anterolateral leg [5]. The lateral cutaneous nerve of calf, which is a branch of the CPN, supplies the upper one-third of the anterolateral leg. The SPN becomes superficial after piercing the fascia above the level of the ankle. It then divides into the intermediate dorsal cutaneous nerve (IDCN) and the medial dorsal cutaneous nerve (MDCN), which provide sensations to the dorsum of the foot except for the first web space that is supplied by the deep peroneal nerve (DPN) [5].

The CPN is most commonly injured at the fibular neck, where the nerve is more superficial just distal to the head of the fibula [4]. However, its branches, DPN and SPN, are more vulnerable to compression distally in the leg, ankle and the foot [6]. Superficial peroneal neuropathy is one of the most frequent complications of foot and ankle arthroscopy [7]. Although neuropathies of the distal branches of the SPN are relatively rare [8], they are prone to injury at the level of ankle or dorsum of the foot by iatrogenic procedures [9]. SPN abnormalities are known to be rarely present in isolation [10-11]. Numerous causes of peroneal neuropathy have been reported. Common etiologies include musculoskeletal injury, compression of the nerve, mass lesions, metabolic syndromes, fractures and ligamentous injuries at knee or ankles. Iatrogenic causes include traction or direct injury of the nerve during surgical exploration, injection procedures, arthroscopy and arthrodesis [9]. Approximately 1% of the peroneal nerve injuries are related to the tibial plateau fracture [4].

Clinical features due to a peroneal nerve injury can vary depending on the location of the injury. Symptoms limited to tingling, numbness and/or pain over the dorsum of the foot except for the first web space [12-13] indicate an involvement of the dorsal cutaneous branches of the SPN. The presence of a foot drop (loss of dorsiflexion of the foot) with the loss of eversion at the ankle joint indicates injury to the DPN or CPN. The loss of sensations to the anterolateral aspect of the lower leg indicate the entrapment of the SPN in the calf [14]. Our patient had paresthesias limited to the lateral aspect of the dorsum of the left foot, indicating the involvement of the DCPN. 

Electrodiagnostic studies consisting of EMG and NCS can be used to confirm the clinical findings. Antidromic evaluation of the sensory branches of the SPN at the ankle is done by placing recording electrodes over the intermediate and medial DCN [15]. These studies are also useful to determine the level and type of the neuronal injury (axonal or demyelinating) [5].

Our patient did not have any prior history of fractures or injuries. Interestingly, the EMG and NCS were normal. The distal branches of the SPN are small and hence are not easily accessible for electrodiagnostic studies [16]. Consequently, these studies can miss the lesions involving the distal branches of the SPN. Persistent symptoms and clinical findings in our patient led us to consider the neuromuscular ultrasound that was remarkable for the enlarged and inflamed dorsal cutaneous nerve, leading to the diagnosis of the entrapment neuropathy at the level of the ankle joint. 

## Conclusions

Although rare, neuropathy involving DCPN can occur. It needs to be suspected in patients presenting with dorsal foot paresthesias, even in non-traumatic settings, with no history of ankle fracture or surgery. Electrodiagnostic studies including EMG and NCS can be inconclusive or normal. In such cases, evaluation with neuromuscular ultrasound can be of diagnostic value.
